# Colorectal cancer screening and incidence and mortality of colorectal and other cancers in the United Kingdom

**DOI:** 10.1111/joim.70075

**Published:** 2026-02-20

**Authors:** Marko Mandic, Fatemeh Safizadeh, Michael Hoffmeister, Hermann Brenner

**Affiliations:** ^1^ Cancer Prevention Graduate School German Cancer Research Center (DKFZ) Heidelberg Germany; ^2^ Division of Clinical Epidemiology of Early Cancer Detection German Cancer Research Center (DKFZ) Heidelberg Germany

**Keywords:** colorectal cancer, fecal‐occult blood test, FOBT, screening, UK Biobank, United Kingdom

Dear Editor,

Randomized controlled trials (RCTs) have demonstrated that colorectal cancer (CRC) screening, including screening by fecal occult blood tests (FOBTs), reduces CRC incidence and mortality under trial conditions. A 2019 meta‐analysis of RCTs found that screening by annual guaiac‐based FOBTs (gFOBTs) was associated with a 31% decrease in CRC mortality (95% CI: −44% to −20%), and a nonsignificant 14% reduction in CRC incidence (95% CI: −28% to +3%) [1]. Although RCTs are crucial for establishing the efficacy of screening offers under trial conditions, additional evidence is needed to evaluate the effectiveness of screening under real‐life conditions. Large‐scale observational epidemiological studies comparing cancer incidence and mortality between screening users and nonusers under real‐life conditions may serve that purpose. However, such studies may be subject to residual confounding factors associated with screening behavior, even if every effort is made to adjust for potential confounders. One way of addressing these concerns is to examine whether the associations with CRC screening are specific to CRC incidence and mortality [2]. If similar associations appear for other cancers that share similar confounding factors but would not plausibly benefit from CRC screening, this would suggest residual confounding.

Therefore, we aimed to assess the associations between CRC screening and CRC incidence and mortality and to compare these associations with the associations with incidence and mortality of other cancers in a very large cohort from the United Kingdom, where organized gFOBT screening began in 2006, initially limited to individuals aged 60–69 years [3].

We analyzed data from 342,164 individuals aged 50–69 years recruited to the UK Biobank [4] between 2006 and 2010. Details of the study population selection, baseline characteristics, and statistical analysis are provided in the Supplement. CRC screening was defined as (self‐reported) ever use of CRC screening, which at the time of recruitment primarily consisted of gFOBTs in the United Kingdom. Associations with CRC incidence and mortality and with anatomic subsites were ascertained using multivariable Cox models and compared with those for other cancers. During a median follow‐up of 11.7 years, 5391 participants were diagnosed with CRC, 39,503 with other cancers, and 1465 died from CRC and 13,465 from other cancers (Supporting Information).

CRC screening was associated with a 14% (95% CI: 10%–18%) lower CRC incidence and a 26% (95% CI: 18%–34%) lower CRC mortality (Fig. 1). No associations were observed for other cancers. Particularly strong associations were seen with distal colon cancer incidence and mortality, with 30% (95% CI: 22%–37%) and 44% (95% CI: 27%–58%) lower risk among screening users, respectively. However, no significant associations were found for proximal colon cancer. These subsite‐specific findings support the results from previous studies and may be attributed to the distinct biological characteristics of proximal versus distal colon lesions. Proximal cancers more often arise from serrated polyps, which are associated with less bleeding and pose greater challenges for detection and removal [5]. The higher proportion of distal colon and rectal cancers among men compared to women could also explain why the reductions in overall CRC incidence and mortality associated with CRC screening are stronger in men.

**Fig. 1:**
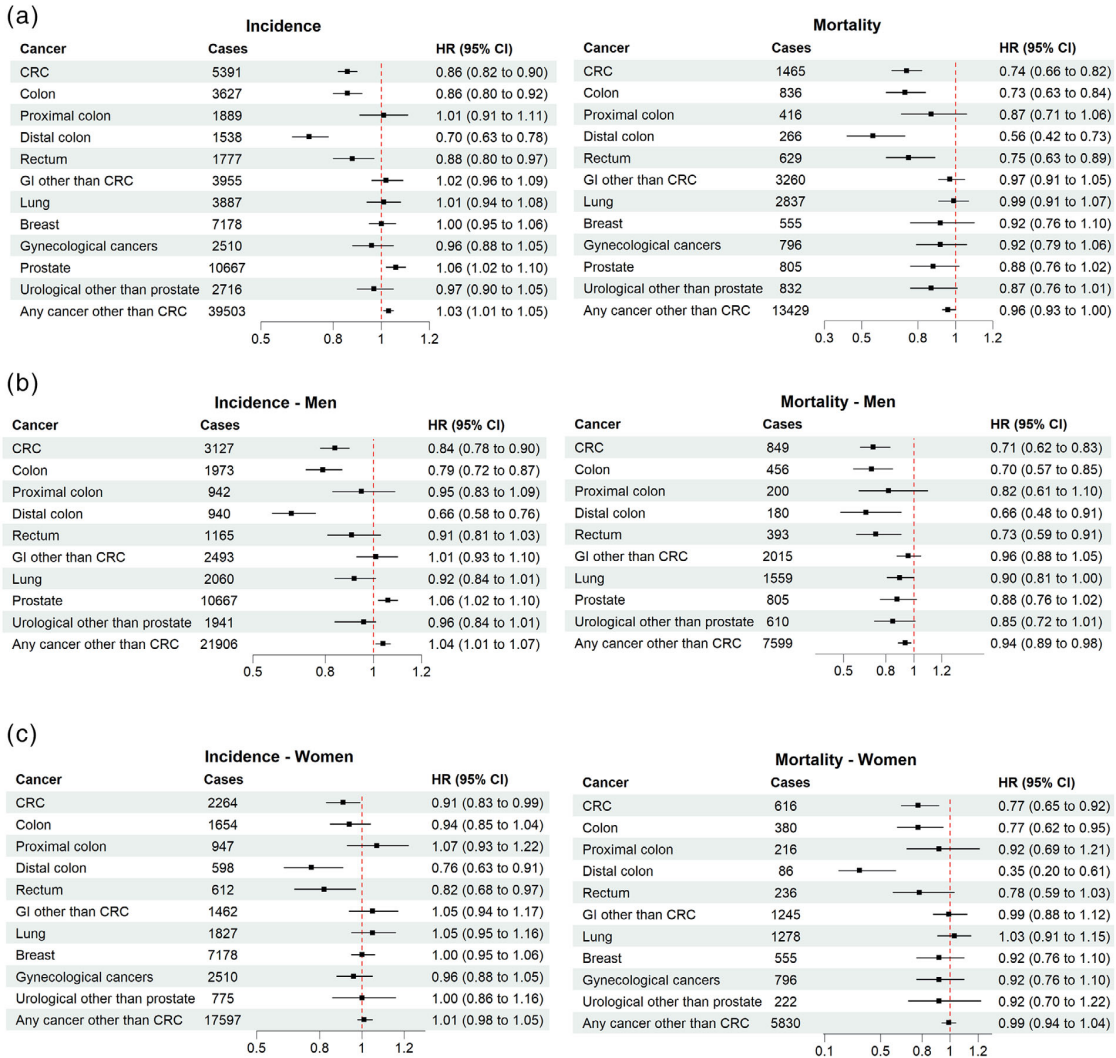
Associations of colorectal cancer screening with incidence and mortality of overall and site‐specific colorectal cancer, other common cancers, and any cancer type other than colorectal cancer. Panel a—total population; Panel b—men; Panel c—women. All cancer types—adjusted for age, sex, ethnic background, education, socio‐economic deprivation, smoking status, alcohol consumption, body mass index, physical activity, fruit and vegetable intake, red and processed meat intake, fish intake, history of CRC in a first‐degree relative, diabetes, hormone replacement therapy (women only), and use of nonsteroidal anti‐inflammatory drugs (NSAIDs). Lung cancer—additionally adjusted for history of lung cancer in a first‐degree relative. Breast cancer—additionally adjusted for age at menarche, parity, menopausal status, history of breast cancer in a first‐degree relative, history of mammography, and use of oral contraceptive pills; assessed in female participants only. Gynecological cancers—additionally adjusted for age at menarche, parity, menopausal status, history of breast cancer in a first‐degree relative, history of Pap test, and use of oral contraceptive pills; assessed in female participants only. Prostate cancer—additionally adjusted for history of prostate cancer in a first‐degree relative and history of prostate‐specific antigen test; assessed in male participants only. Any cancer other than CRC—additionally adjusted for family history of breast, prostate, and lung cancers. CI, confidence interval; CRC, colorectal cancer; HR, hazard ratio.

Major strengths of our analysis include the prospective design, large sample size, extended follow‐up, and comprehensive adjustment for potential confounders. However, despite careful control for known confounders, we cannot fully dismiss the possibility of residual confounding. Moreover, use of CRC screening was self‐reported and included only ever use at baseline, with no information on subsequent uptake during follow‐up. This likely led to underestimation of the associations with reductions in CRC incidence and mortality compared to participation in an organized screening program with biennial invitations. Additionally, the predominantly White, health‐conscious UK Biobank population limits generalizability.

In England, the age‐standardized incidence rate of CRC remained approximately the same between 2000 and 2017, whereas CRC mortality decreased significantly (average annual percentage change of about −1.5%) [6]. This decline in CRC mortality is likely a direct result of the shift to earlier stage diagnosis achieved through the national CRC screening programme [7]. Additionally, CRC screening uptake has increased substantially over the past decade, with 2.5‐year coverage reaching 70.3% in 2022 [8]. Since 2019, gFOBT has been replaced by a more sensitive and easier‐to‐use faecal immunochemical test (FIT) [9]. Taking these factors into account, it is likely that the reduction in CRC mortality in England will continue.

This large, prospective cohort study in the United Kingdom provides crucial real‐world evidence on the effectiveness of FOBT‐based CRC screening in reducing CRC incidence and mortality, complementing and corroborating the existing evidence from RCTs. These findings underscore the effectiveness of CRC screening as a preventive measure and support ongoing efforts to implement effective CRC screening programmes and foster their use. As CRC screening guidelines continue to evolve, future evaluations of cancer screening effectiveness in both trial and real‐world settings will be critical for informing policy decisions and optimizing screening protocols.

## Author contributions

Marko Mandic and Fatemeh Safizadeh contributed to the conception and design of the study, data analysis, interpretation of results, and drafting of the manuscript. They contributed equally to this work and share first authorship. Hermann Brenner supervised the study and contributed to its conception and design, interpretation of findings, drafting, and critical revision of the manuscript. Michael Hoffmeister contributed to the interpretation of the findings and critically revised the manuscript for important intellectual content. All authors read and approved the final version of the manuscript.

## Conflict of interest statement

The authors declare no conflicts of interest.

## Funding information

UK Biobank was established by the Wellcome Trust medical charity, Medical Research Council, Department of Health, Scottish Government and the Northwest Regional Development Agency. It has also had funding from the Welsh Government, British Heart Foundation, Cancer Research UK, Diabetes UK, and National Institute for Health Research (NIHR). UK Biobank is supported by the National Health Service (NHS).

## Ethics statement

The UK Biobank was approved by the North West Multi‐centre Research Ethics Committee (MREC) as a Research Tissue Bank (RTB) approval (renewed approval in 2021:21/NW/0157).

## Consent

Electronic informed consent was obtained from all individual participants included in the UK Biobank.

## Supporting information




**Supplementary Figure 1**: Flowchart of the selection of study population.
**Supplementary Table 1**: Distribution of baseline characteristics of the study participants, according to colorectal cancer screening.
**Supplementary Table 2**: List of cancer types (ICD‐10) included in the analysis.

## Data Availability

Data were reused with the permission of the UK Biobank. This work used data provided by patients and collected by the NHS as part of their care and support. The UK Biobank is an open‐access resource, and bona fide researchers can apply to use the UK Biobank dataset by registering and applying at https://www.ukbiobank.ac.uk/enableyourresearch/apply‐for‐access. The data and analysis codes used for this study will be available on the UK Biobank website for registered researchers at the UK Biobank and an application fee.
