# Supplementary material for: Colorectal cancer screening and incidence and mortality of colorectal and other cancers in the United Kingdom
Source: J Intern Med. 2026 Feb 20;299(5):639–42. doi: 10.1111/joim.70075 (PMC13061097; doi:10.1111/joim.70075)
Supplement: Supplementary file 1 — Supplementary Figure 1: Flowchart of the selection of study population. Supplementary Table 1: Distribution of baseline characteristics of the study participants, according to colorectal cancer screening. Supplementary Table 2: List of cancer types (ICD‐10) included in the analysis. [file JOIM-299-639-s001.docx]

**Supplementary material**

**Colorectal cancer screening and incidence and mortality of colorectal and other cancers in the United Kingdom**

**Supplement Text – Methods**

**Supplementary Figure 1.** Flowchart of the selection of study population.

**Supplementary Table 1.** Distribution of baseline characteristics of the study participants, according to colorectal cancer screening.

**Supplementary Table 2.** List of cancer types (ICD-10) included in the analysis.

## Supplement Text - Methods

## Study population

This study utilized data from the UK Biobank, a large prospective cohort that includes approximately 500,000 participants from England, Wales, and Scotland, aged between 40 and 69 years at the time of recruitment (2006–2010). Detailed information about the UK Biobank study has been published previously.^1^ The UK Biobank has collected extensive socio-demographic, environmental, and lifestyle data through self-administered touch-screen questionnaires and computer-assisted interviews. It is also linked to national registries and electronic health records, and, therefore, includes data on cancer, death, and primary care. The UK Biobank has obtained ethical approval from the North West Multi-centre Research Ethics Committee (MREC) as a Research Tissue Bank (RTB), which was renewed in 2021 (21/NW/0157). All UK Biobank participants provided signed informed consent. In this study, the analysis was limited to participants aged 50 years and older, as this is the minimum recommended age for bowel cancer screening in average-risk individuals across various regions in the UK. For both the cancer incidence and mortality outcomes, participants with a cancer diagnosis (except non-melanoma skin cancer) before or at recruitment, as well as those with missing CRC screening data were excluded from the analysis.

## Exposure ascertainment

CRC screening was defined as (self-reported) ever use of colorectal cancer screening, which at the time of recruitment primarily consisted of guaiac-based fecal occult blood tests (gFOBTs) in the UK.

## Cancer incidence

Cancer data were obtained from the UK Biobank through linkage with national cancer registries. Cancer incidence was classified according to the International Statistical Classification of Diseases (ICD-10). CRC and other cancers specifically addressed in the analyses are detailed in **Supplementary** **Table 1**. At the time of analysis, complete follow-up data were available until December 31, 2020, for England; November 30, 2021, for Scotland; and December 31, 2016, for Wales.

## Cancer-specific mortality

The primary cause of death was determined through linkage with national death registries. Cancer-specific mortality for various cancer types was classified using ICD-10 codes. At the time of analysis, complete mortality data were available for all regions up until November 30, 2022.

## Statistical Analysis

Descriptive statistics were used to summarize the baseline characteristics of the cohort. The associations of CRC screening with cancer incidence and mortality were evaluated using Cox proportional hazards models. For cancer incidence, the follow-up period was established from the initial assessment visit to the earliest occurrence of a specified cancer diagnosis, death, loss to follow-up, or the censoring date. For cancer-specific mortality, the follow-up period was calculated from the initial assessment visit to the occurrence of cancer-specific death, death from any other causes, loss to follow-up, or the censoring date, whichever came first. For both outcomes, we performed analyses using two models: the first model was adjusted for age at baseline (years) and sex (male, female), and the second model (fully adjusted model) was adjusted for additional covariates which were pre-selected as potential confounders based on previous knowledge about their association with CRC risk: self-reported ethnic background (white, other), socioeconomic status (Townsend deprivation index), educational qualifications (higher academic/professional, lower academic/vocational, or none), body mass index (kg/m^2^), pack-years of smoking (years), alcohol consumption (never, special occasions only, 1-3 times a month, once or twice a week, 3-4 times a week, daily or almost daily), level of physical activity determined by the International Physical Activity Questionnaire (IPAQ)^2^ (low, moderate, high), self-reported average intake of fruit (fresh and dried; pieces/day), vegetable (raw and cooked; tablespoons/day), wholegrains (bran cereal, oat cereal, whole meal or wholegrain bread; servings/week), refined grains (biscuit cereal, muesli, cornflakes, Frosties, white and brown bread; servings/week), fish (oily and non-oily; never, less than once a week, once a week, ≥2 times a week), and red (beef, lamb, pork) and processed meat (never, less than once a week, once a week, ≥2 times a week), diabetes (non-diabetic, diabetic), history of CRC in a first-degree relative, and regular use of non-steroidal anti-inflammatory drugs (NSAIDs), hormone-replacement therapy (HRT) (women only).

For comparison, analogous analyses were conducted to assess the association of CRC screening with incidence and mortality of the following other common cancers: gastrointestinal other than CRC, lung, breast (women only), gynecological, prostate, urological other than prostate, and any cancer other than CRC. The fully adjusted model for breast cancer was adjusted for age at menarche (years, ≤12, 13-14, >14), parity (0, 1, 2, ≥3), menopausal status (pre-menopausal, post-menopausal), history of breast cancer in a first-degree relative, history of mammography, and use of oral contraceptive pills (OCPs), along with the variables accounted for in the fully adjusted model for other cancers. The fully adjusted model for gynecological cancers included all covariates included in the fully adjusted model for breast cancer but was adjusted for Pap test instead of mammography. The fully adjusted model for prostate cancer was adjusted for history of prostate cancer in a first-degree relative, and history of prostate-specific antigen (PSA) test, in addition to the covariates included in the fully adjusted model for other cancer types. The models for breast and gynecological cancers were restricted to female participants, and the model for prostate cancer was restricted to male participants.

To address missing covariate values, multiple imputation was carried out using PROC MI in SAS software, version 9.4 (SAS Institute Inc).^3^ All variables included in the main analysis were also included in the imputation procedure (100 iterations and 5 imputed datasets). The analyses were then conducted with five imputed datasets using the SAS PHREG procedure, and the results were pooled using PROC MIANALYZE. Notably, physical activity data had approximately 20% missing values, while all other covariates had less than 2% missing data, and there were no missing values for age and sex. The proportional hazards assumption was examined using Schoenfeld residual plots, and no violations were detected.

All statistical analyses were conducted using SAS version 9.4 and R version 4.3.2^4^ (R Project for Statistical Computing). All statistical tests were two-sided and p-value less than 0.05 was considered significant.

Supplementary Text References

1. Sudlow C, Gallacher J, Allen N, et al. UK Biobank: An Open Access Resource for Identifying the Causes of a Wide Range of Complex Diseases of Middle and Old Age. *PLOS Med*. 2015;12(3):e1001779. doi:10.1371/journal.pmed.1001779

2. Booth M. Assessment of physical activity: an international perspective. *Res Q Exerc Sport*. 2000;71 Suppl 2:114-120. doi:10.1080/02701367.2000.11082794

3. Little RJA, Rubin DB. *Statistical Analysis with Missing Data*. John Wiley & Sons; 2019.

4. R Core Team. R: A language and environment for statistical computing. Published online 2022. https://www.R-project.org/

N= 499,926 UK Biobank cohort (40-69 years)

*n*=117,801 Had Age<50 years

N= 382,125 UK Biobank cohort (50-69 years)

*n*=33,316 Had history of cancer diagnosis (except non-melanoma skin cancer)

*n*=6,645 Had missing colorectal cancer screening information

N=342,164 Participants included in analysis

**Supplementary Figure 1.** Flowchart of the selection of study population.

| **Variable** | **All Participants** | **CRC Screening** | **No CRC Screening** |
| --- | --- | --- | --- |
| **Age at recruitment** |  |  |  |
| 50-59 | 152,495 (44.6) | 28,884 (23.1) | 123,611 (56.9) |
| 60-69 | 189,669 (55.4) | 96,158 (76.9) | 93,511 (43.1) |
| Median (IQR), years | 60 (55-64) | 63 (60-66) | 58 (54-63) |
| **Sex** |  |  |  |
| Female | 182,742 (53.4) | 64,446 (51.5) | 118,296 (54.5) |
| Male | 159,422 (46.6) | 60,596 (48.5) | 98,826 (45.5) |
| **Ethnicity** |  |  |  |
| White | 326,752 (95.8) | 120,510 (96.7) | 206,242 (95.3) |
| Other | 14,237 (4.2) | 4,095 (3.3) | 10,142 (4.7) |
| **Deprivation Index, Median (IQR)** | -2.3 (-3.7-0.3) | -2.3 (-3.7-0.2) | -2.3 (-3.7-0.3) |
| **Educational Qualifications** |  |  |  |
| Higher academic/professional | 160,662 (47.5) | 57,531 (46.5) | 103,131 (48.0) |
| Lower academic/vocational | 108,154 (32.0) | 38,389 (31.1) | 69,765 (32.5) |
| None | 69,626 (20.6) | 27,732 (22.4) | 41,894 (19.5) |
| **Body mass index (kg/m^2^)** |  |  |  |
| <25 | 106,892 (31.4) | 38,171 (30.7) | 68,721 (31.8) |
| 25-<30 | 148,134 (43.5) | 55,213 (44.4) | 92,921 (43.0) |
| ≥30 | 85,371 (25.1) | 31,039 (25.0) | 54,332 (25.2) |
| Median (IQR) | 26.9 (24.3-30.0) | 26.9 (24.4-30.0) | 26.9 (24.3-30.0) |
| **Pack-years of smoking (years)** |  |  |  |
| 0 | 182,229 (63.3) | 64,025 (61.3) | 118,204 (64.4) |
| >0-20 | 52,407 (18.2) | 19,888 (19.0) | 32,519 (17.7) |
| >20-40 | 35,221 (12.2) | 13,308 (12.7) | 21,913 (11.9) |
| ≥40 | 18,165 (6.3) | 7,281 (7.0) | 10,884 (5.9) |
| **Alcohol consumption** |  |  |  |
| Never | 27,432 (8.0) | 10,125 (8.1) | 17,307 (8.0) |
| Special occasions only | 39,225 (11.5) | 14,456 (11.6) | 24,769 (11.4) |
| One to three times a month | 35,447 (10.4) | 12,417 (9.9) | 23,030 (10.6) |
| Once or twice a week | 85,038 (24.9) | 29,554 (23.7) | 55,484 (25.6) |
| ≥three times a week | 154,772 (45.3) | 58,421 (46.8) | 96,351 (44.4) |
| **Physical activity (IPAQ groups)** |  |  |  |
| Low | 47,697 (18.3) | 16,785 (17.6) | 30,912 (18.8) |
| Moderate | 106,782 (41.0) | 39,398 (41.3) | 67,384 (40.9) |
| High | 105,686 (40.6) | 39,208 (41.1) | 66,478 (40.3) |
| **Fruit intake (pieces/day)** |  |  |  |
| <3 | 162,051 (47.4) | 56,914 (45.6) | 105,137 (48.5) |
| ≥3 | 179,513 (52.6) | 67,947 (54.4) | 111,566 (51.5) |
| **Vegetable intake (tablespoons/day)** |  |  |  |
| <3 | 56,620 (16.7) | 18,978 (15.3) | 37,642 (17.5) |
| ≥3 | 283,475 (83.4) | 105,388 (84.7) | 178,087 (82.6) |
| **Red meat intake** |  |  |  |
| Never | 20,656 (6.1) | 6,836 (5.5) | 13,820 (6.4) |
| Less than once a week | 126,184 (37.2) | 46,175 (37.3) | 80,009 (37.2) |
| Once a week | 73,118 (21.6) | 26,826 (21.6) | 46,292 (21.5) |
| ≥2 times a week | 119,005 (35.1) | 44,114 (35.6) | 74,891 (34.8) |
| Continues | | | |

**Supplementary Table 1.** Distribution of baseline characteristics of the study participants, according to colorectal cancer screening.

| **Characteristics** | **All Participants** | **FOBT Screening** | **No FOBT Screening** |
| --- | --- | --- | --- |
| **Processed meat intake** |  |  |  |
| Never | 30,916 (9.1) | 10,613 (8.5) | 20,303 (9.4) |
| Less than once a week | 106,807 (31.3) | 39,054 (31.3) | 67,753 (31.3) |
| Once a week | 100,449 (29.4) | 36,641 (29.4) | 63,808 (29.5) |
| ≥2 times a week | 103,303 (30.3) | 38,494 (30.8) | 64,809 (63.5) |
| **Fish intake** |  |  |  |
| Never | 12,947 (3.8) | 3,915 (3.4) | 9,032 (4.2) |
| Less than once a week | 59,562 (17.4) | 19,702 (15.8) | 39,860 (18.4) |
| Once a week | 83,567 (24.5) | 29,683 (23.8) | 53,884 (24.9) |
| ≥2 times a week | 185,527 (54.3) | 71,567 (57.3) | 113,960 (52.6) |
| **Wholegrains (servings/week), Median (IQR)** | 1.0 (0.0-2.0) | 1.0 (0.0-2.0) | 1.0 (0.0-2.0) |
| **Refined grains (servings/week), Median (IQR)** | 0.9 (0.0-1.7) | 0.9 (0.0-1.7) | 0.9 (0.0-1.7) |
| **Family history of CRC** |  |  |  |
| No | 295,547 (88.0) | 103,464 (84.2) | 192,083 (90.1) |
| Yes | 40,436 (12.0) | 19,378 (15.8) | 21,058 (9.9) |
| **Diabetes** |  |  |  |
| Non-diabetic | 320,808 (94.0) | 116,480 (93.4) | 204,328 (94.3) |
| Diabetic | 20,500 (6.0) | 8,226 (6.6) | 12,274 (5.7) |
| **Use of NSAIDs** |  |  |  |
| No | 233,412 (68.3) | 83,343 (66.7) | 150,069 (69.2) |
| Yes | 108,546 (31.7) | 41,638 (33.3) | 66,908 (30.8) |
| **Use of HRT (women only)** |  |  |  |
| No | 95,348 (52.3) | 27,862 (43.4) | 67,486 (57.2) |
| Yes | 86,866 (47.7) | 36,402 (56.6) | 50,464 (42.8) |

Supplementary Table 1 (continued)

Missing values (N): Age (0), sex (0), ethnic background (1,175), Townsend deprivation index (363), educational qualifications (3,722), body mass index (1,767), pack-years of smoking (54,142), alcohol consumption (250), physical activity (81,999), fruit intake (600), vegetable intake (2,069), red meat consumption (3,201), processed meat intake (689), fish intake (561), wholegrain intake (3,918), refined grain intake (4,362), CRC family history (6,181), diabetes (856), use of NSAIDs (206), HRT use (528).

Data are expressed as number of participants (percentage) unless otherwise specified. Percentages might not add up to 100 percent due to rounding.

Abbreviations: CRC: Colorectal cancer; HRT: Hormone replacement therapy; IPAQ: International Physical Activity Questionnaire; IQR: Interquartile range; NSAIDs: Nonsteroidal anti-inflammatory drugs.

**Supplementary Table 2.** List of cancer types (ICD-10) specifically addressed in the analysis.

| **Cancer site** | **ICD-10 code** |
| --- | --- |
| Colon and rectum | C18.0- C18.9, C19, C20 |
| Colon | C18.0- C18.9 |
| Proximal colon | C180, C182, C183, C184 |
| Distal colon | C185, C186, C187 |
| Rectum | C19, C20 |
| GI other than colon and rectum | Esophagus: C15  Stomach: C16  Small intestine: C17  Liver: C22  Pancreas: C25  Gallbladder: C23  Bile duct: C24  Anus: C21.0-C21.2 |
| Lung | C34 |
| Breast | C50 |
| Gynecological tract | Cervix: C53  Ovary: C56  Endometrium: C54  Vagina: C52  Vulva: C51  Other: C57 |
| Prostate | C61 |
| Urological tract other than prostate | Kidney: C64  Renal pelvis: C65  Bladder: C67  Urethra: C68.0  Ureter: C66  Testis: C62  Penis: C60  Other: C68.1, C68.8-C68.9 |
| Any cancer except CRC (excluding non-melanoma skin cancer) | C00-C97 excluding C44, C18.0- C18.9, C19, C20 |

Abbreviations: CRC: Colorectal cancer; ICD-10: International Statistical Classification of Diseases 10^th^ revision; GI: Gastrointestinal tract.
